# Bovine mastitis: risk factors, therapeutic strategies, and alternative treatments — A review

**DOI:** 10.5713/ajas.20.0156

**Published:** 2020-05-12

**Authors:** Wei Nee Cheng, Sung Gu Han

**Affiliations:** 1 Department of Food Science and Biotechnology of Animal Resources, Konkuk University, Seoul 05029, Korea

**Keywords:** Bovine Mastitis, Dairy Cow, Bacteria, Antibiotic, Alternative Bovine Mastitis Treatment, Natural Products

## Abstract

Bovine mastitis, an inflammation of the mammary gland, is the most common disease of dairy cattle causing economic losses due to reduced yield and poor quality of milk. The etiological agents include a variety of gram-positive and gram-negative bacteria, and can be either contagious (e.g., *Staphylococcus aureus*, *Streptococcus agalactiae*, *Mycoplasma* spp.) or environmental (e.g., *Escherichia coli*, *Enterococcus* spp., coagulase-negative *Staphylococcus*, *Streptococcus uberis*). Improving sanitation such as enhanced milking hygiene, implementation of post-milking teat disinfection, maintenance of milking machines are general measures to prevent new cases of mastitis, but treatment of active mastitis infection is dependant mainly on antibiotics. However, the extensive use of antibiotics increased concerns about emergence of antibiotic-resistant pathogens and that led the dairy industries to reduce the use of antibiotics. Therefore, alternative therapies for prevention and treatment of bovine mastitis, particularly natural products from plants and animals, have been sought. This review provides an overview of bovine mastitis in the aspects of risk factors, control and treatments, and emerging therapeutic alternatives in the control of bovine mastitis.

## INTRODUCTION

Bovine mastitis is an inflammatory response of the udder tissue in the mammary gland caused due to physical trauma or microorganism infections. It is considered the most common disease leading to economic loss in dairy industries due to reduced yield and poor quality of milk [1]. On an average, the total failure cost due to bovine mastitis is estimated to be $147 per cow per year, particularly contributed by milk production losses and culling, which represents 11% to 18% of the gross margin per cow per year [2]. Mammary tissue damage leading to decreased milk production accounts for 70% of the total losses [3].

Bovine mastitis can be classified into 3 classes based on the degree of inflammation, namely clinical, sub-clinical, and chronic mastitis. A clinical bovine mastitis is evident and easily detected by visible abnormalities, such as red and swollen udder, and fever in dairy cow. The milk of the cow appears watery with presence of flakes and clots [4]. Clinical mastitis can be further sub-divided into per-acute, acute, and sub-acute depending on degree of the inflammation [5]. Severe cases of clinical mastitis can also be fatal [6]. Contrary to clinical mastitis, sub-clinical mastitis shows no visible abnormality in the udder or milk, but milk production decreases with an increase in the somatic cell count (SCC) [7]. The loss contributed by sub-clinical mastitis is very hard to quantify, but experts agree that it accounts for more financial losses in the herd than do clinical cases [3,8]. Contrarily, chronic mastitis is an inflammatory process that lasts for several months, with clinical flare-ups occurring at irregular intervals.

This review article provides an overview of risk factors that are known to be associated with the incidence and severity of bovine mastitis. In spite of the fact that the use antibiotic remains as the main treatment strategy for bovine mastitis, concerns about emergence of antibiotic-resistance pathogens are continuously raising. In this regard, this review will highlight some therapeutic alternatives to antibiotic that may control bovine mastitis. The recent publications about the natural compounds derived from plants, animals, and bacteria will be discussed and their mechanisms of action also will be described in this review.

## BOVINE MASTITIS RISK FACTORS

There are several risk factors known to be associated with the incidence of bovine mastitis that play significant role, including pathogen, host, and environmental factors. All these factors were taken into consideration in the mastitis control programs [9].

### Pathogen factor

Bacterial intra-mammary infection (IMI) is considered to be the main cause of bovine mastitis. Many bacterial species have been identified as causative agents for bovine mastitis. These bacterial infections can be classified into 2 types based on the bacterial origin—contagious and environmental [10]. Contagious mastitis refers to mastitis that can be transmitted from cow-to-cow, especially during milking [11]. Contagious pathogens such as *Staphylococcus aureus* and *Streptococcus agalactiae*, and less common species like *Mycoplama bovis* and *Corynebacterium*, live on the cow’s udder and teat skin, colonizing and growing into the teat canal [5]. These are capable of establishing sub-clinical infections, usually with an elevation in the SCC. The SCC is a useful indication of IMI infection and that consist of leukocytes (i.e., neutrophils, macrophages, lymphocytes, and erythrocytes) and epithelial cells [12]. Contagious infections can be controlled by reducing contact between reservoirs and uninfected cows. Therefore, proper maintenance of milking equipment, post-milking teat disinfection, culling, and dry cow therapy (DCT) are important to prevent contagious infections [13].

Unlike contagious pathogens, environmental pathogens do not usually live on the cow’s udder and teat skin; instead, they exist in the bedding and housing of the herd. They are best described as opportunistic pathogens, looking for the chance to cause an infection. For example, they can enter the teat during milking owing to the liner slippage, or when cow’s natural immunity is weak, causing clinical mastitis. Environmental pathogens such as *Escherichia coli* (*E. coli*) or *Strep. uberis* invade and multiply in the cow’s udder, induce a host immune response and are rapidly eliminated [14]. A wide range of bacterial species were reported to cause environmental mastitis, namely, *Streptococcus* spp. (e.g. *Strep. uberis*), coliforms species (e.g. *E. coli*, *Klebsiella* spp., *Enterobacter* spp.), *Pseudomonas* spp., etc [15]. Control of environmental infection can be achieved by reducing exposure of teat ends to environmental pathogens and by boosting resistance of the cow to IMI by antibiotic intervention and vaccination [13].

#### Staphylococcus aureus

*Staph. aureus* is the most prevailing gram-positive pathogen known to be associated with various forms of clinical and sub-clinical mastitis [16]. The fundamental reservoir of *Staph. aureus* is chronically infected mammary gland, therefore maintaining hygiene of udder and milking can protect healthy cow from infected cow, thereby reducing the infection [17]. *Staph. aureus* does not trigger an immune response in cow as strong as *E. coli* or endotoxin, therefore the infection of *Staph. aureus* is always milder, leading to chronic mastitis that lasts for a few months [18]. *Staph. aureus* do not cause abnormalities or fatality; however, it produces degradative enzymes and toxins that irreversibly damage the milking tissue, ultimately decreasing milk production [16].

Treatment of *Staph. aureus* infections is done by the use of antibiotics. However, Rainard et al [17] demonstrated that antibiotic is not an efficient method due to resistance developed by the pathogen against β-lactam antibiotics, i.e., methicillin. Such strains of *Staph. aureus* are known as methicillin-resistant *Staph. aureus* (MRSA), which have a *mecA* gene conferring the resistance [19]. Besides that, the ability of *Staph. aureus* to produce biofilm and adapt to host environment makes it an even harder target for treatment of such infection [20,21].

Biofilms are cluster of cells (a structured community of bacterial cells) enclosed in a self-produced matrix (exopolysaccharides, proteins, teichoic acids, enzymes, and extracellular DNA), adherent to biotic or abiotic surfaces [22]. Biofilm formation is initiated with attachment of bacteria to an abiotic surface, which can be driven by hydrophobic or electrostatic interactions followed by adhesion facilitated by cell wall associated adhesins (i.e., flagella, fimbriae, and pili), often involving the formation of polymer bridges between bacteria and the cell surface. After which, bacteria begin to multiply forming a multicellular structure, connected to each other by extracellular polysaccharides. Finally, when the biofilm reaches a critical mass, a dynamic equilibrium is reached at which outer most cell layers begin to generate planktonic organisms. These bacteria are free to escape from the biofilm and migrate and colonize other surfaces [23,24]. *Staph. aureus* produces an exopolysaccharide called glycocalyx. It helps *Staph. aureus* to adhere to mammary epithelial cells and acquire nutrient, allowing it to survive in high shear environment, as well as protect it from environmental stress such as antibiotics, disinfectants, and evade phagocytosis [23,25]. This is the main reason why a gram-positive bacterium is hard to treat using antibiotic, because the antibiotic does not reach the target site at minimum inhibitory concentration (MIC) but only at a sub-MIC which is not able to kill the pathogen [26]. As a result, alternative therapies that directly target biofilm forming ability of *Staph. aureus* are necessary [27].

#### Streptococcus agalactiae

*Strep. agalactiae* is a gram-positive pathogen causing contagious mastitis. It can be found in bovine gastrointestinal tract as well as in the environment of dairy cows. It can be transmitted via milking machine and through oro-fecal route, particularly through contaminated drinking water; therefore, a recent study showed that maintaining udder and milking sanitary are not enough to control *Strep. agalactiae* infection, but fecal and environment management should also be taken into account [28]. *Strep. agalactiae* causes sub-clinical mastitis with high SCC and low milk production even though no abnormalities were shown in milk [5]. It can survive indefinitely in mammary glands of cows, by forming a biofilm that allows them to adhere and persist in the mammary gland, concomitantly enhancing resistance to host factor and nutrient deprivation [29].

#### *Mycoplasma* spp.

Contagious mastitis caused by *Mycoplasma* spp. is less common than *Staph. aureus* and *Strep. agalactiae* infection. However, it is highly severe and damage secretory tissues, and induce gland and lymphatic nodule fibrosis and abscesses [5]. Outbreak of Mycoplasmal mastitis is sporadic without any deliberate intervention. Although it is self-limiting, it produces biofilm and invades host cell, and does not respond to antibiotic treatment [30]. The only control is by regular monitoring and rapid segregation or culling of infected cow [31].

#### Escherichia coli

*E. coli* is the most frequently found gram-negative pathogen. It invades the udder through teat, proliferate and initiate inflammatory response in dairy cow. It can be found in the environment surrounding dairy cow, such as bedding of the herd, especially in a wet condition [13]. Mastitis caused by *E. coli* is usually clinical and transient. Symptoms are varied, ranging from mild with only local signs (red and swollen udder) to severe with systemic signs (fever). Severe clinical mastitis caused by *E. coli* can cause irreversible tissue damage in the mammary gland, complete loss of milk production, sometimes even leading to the death of dairy cow.

*E. coli* rapidly induce an inflammatory response in the host. The virulence factor best known to trigger the inflammatory response is the endotoxin, which is found on the outer membrane of *E. coli*, known as lipopolysaccharide (LPS). The binding of LPS to toll-like receptor (TLR4) in association with other molecules, such as LPS-binding protein and cluster of differentiation 14 induce a series of signaling pathways [32]. TLR4 engagement activates myeloid differentiation factor 88 (MyD88) and recruit members of interleukin-1 receptor-associated kinase family and tumor necrosis factor receptor-associated factor 6, which then activates transforming growth factor β-activated kinase 1 (TAK1) complex. Activated TAK1 complex acts as an inhibitor of nuclear factor-κB kinase (IKK) complex consisting of IKKα, IKKβ, and nuclear factor kappa B (NF-κB) kinase essential modulator (NEMO), which then brings about phosphorylation of inhibitor of NF-κB (IκB). Degradation of IκB releases NF-κB and translocate it into the nucleus. Simultaneously, TAK1 also brings about phosphorylation of mitogen-activated protein kinases (MAPK) such as extracellular signal-regulated kinase (ERK), c-JUN N-terminal kinase, and p38 mitogen-activated protein kinase (p38), that results in nuclear translocation of activator protein 1 [33,34]. NF-κB is an important protein in the complex that control DNA transcription, cytokine production and cell survival. The binding of NF-κB to the DNA sequence results in transcription of mRNA and translation of inflammatory cytokines such as TNF-α, interleukin (IL)-1β, IL-6, IL-8, and inflammatory markers such as cyclooxygenase-2 (COX-2), inducible nitric oxide synthase (iNOS), ultimately leading to an inflammatory response [35]. Pro-inflammatory cytokines not only play a role in initiating inflammatory responses at both local and systemic level, but also activate and enhance the functions of leukocytes such as neutrophils and macrophages by migrating to the target sites, and clearing infections [18,36]. The host defense status act as a pivotal factor in determining the outcome of infections. In view of this, the severity of *E. coli* mastitis is mainly determined by the host factor rather than by *E. coli* pathogenicity [33].

Nevertheless, *E. coli* was classified as an opportunistic pathogen with different virulence factors, since its pathogenicity is not only mediated by single and specific virulence factor [37]. In fact, combinations of several virulence factors such as toxins, adhesins, invasins, capsule production, ability to resist serum complement, and iron scavenging, are reported as being necessary to overcome the host’s selection pressure and to colonize, multiply, and survive in the udder and cause inflammatory responses [38]. Besides, *E. coli* can persist in the mammary gland, causing recurrent mastitis infections that are hard to treat, possibly due to the ability to produce biofilm at different levels [1,37].

#### *Enterococcus* spp.

*Enterococcus faecalis* is the predominant *Enterococcus* spp., followed by *Ent. faecium*. They are environmental gram-negative pathogens present in the organic bedding material of the herd. Pathogenesis of *Ent. faecalis* was reported related to the biofilm formation [39]. In addition, both *Ent. faecalis* and *Ent. faecium* were reported to be resistant to several antibiotics such as lincomycin, tetracycline, kanamycin, streptomycin, quinupristin/dalfopristin (Synercid), erythromycin, chloramphenicol, and tylosin owing to the presence of biofilm [40]. This leads to frequent occurrences of enterococci infections, both recurrent and persistent, which are difficult to treat [39].

#### Coagulase-negative Staphylococcus

Coagulase-negative *Staphylococcus* (CNS), for example *Staph. simulans*, *Staph. chromogens*, *Staph. hyicus*, and *Staph. epidermis*, represents an emerging mastitis pathogen that has been isolated in many countries. The infections caused by CNS are relatively mild, usually remain sub-clinical but can be persistent, and are associated with an elevated SCC and decreased milk quality [41]. However, unlike *Staph. aureus*, reports show that their persistence in the udder has no relation with the ability of biofilm production [42]. They can behave as both contagious and environmental pathogens. So, post-milking teat disinfection is an effective measure in reducing CNS infections; as well as antibiotic intervention. CNS responds better to antibiotic treatment than *Staph. aureus* [41].

#### Streptococcus uberis

*Strep. uberis* is an environmental pathogen that causes recurrent mastitis, associated with clinical and sub-clinical infections [43]. It was reported that α-casein and β-casein component in milk induce production of biofilm, which help *Strep. uberis* to persist under environmental stress and resist antibiotic treatment [1,44]. It has been detected in different part of animals including lips, tonsils, skin, oral cavity, rumen, respiratory tract, rectum, teat orifice, teat canals, infected udders, feces, and wounds [45].

### Host factor

#### Breeding and genetic

Genetic factors and dairy cow breeding have an effect on susceptibility or resistance to mastitis. Pure breed or cross breed of high-yielding cattle, particularly, Holstein-Friesian cattle, appear to be more genetically vulnerable to mastitis than are breeds giving medium yield [46]. For instance, Jersey cattle were reported to have lower rate of mastitis than Holstein-Friesian cattle [47]. In addition, lower-yielding Rendena cattle which are native to north-eastern Italy, demonstrated higher resistance and resilience to diseases including mastitis [48]. Moreover, multiparous cows are more vulnerable to IMI than primiparous cows due to immunoincompetence [46].

#### Udder structure

The structure of the udder also affects the susceptibility to the infection. Cattle with large funnel-shaped teats or pendular-shaped udder and blind quarters after calving are at greater risk of sub-clinical mastitis [49]. Other than that, teat size and teat to floor distance may also decrease the *in vitro* activity of leukocytes in milk hence increase the occurrence of IMIs [50].

#### Age

Another factor influencing infections is age. Older cow are more susceptible to infections, most probably because of the wider or permanently partially-open teat canal as a results of frequent milking [5]. Furthermore, mammary epithelium of older cow has increased permeability, mainly because of the irreversible damage caused by previous inflammations [51].

#### Transition period

The period between 3 weeks before and after parturition is defined as transition period, also known as periparturient period. Dairy cows are at a higher risk to acquire diseases like mastitis during this period [52]. Researchers showed that IMIs occur more at parturition and first month of lactation [53,54]. The high incidence of mastitis was reported due to immunosuppression, associated with the increased oxidative stress and low antioxidant defense [7,55].

#### Host nutritional stress and immune system

During lactation, there is a higher demand of energy and nutrient for the synthesis of colostrum and milk by the dairy cattle. So, when the feed intake does not meet the lactation demands, cattle exhibit negative energy balance [5]. Negative energy balance is associated with the diet deficiencies in trace elements (i.e., selenium, iron, copper, zinc, cobalt, chromium), amino acids (i.e., lysine, L-histidine), and vitamins (i.e., A, C, E, β-carotene, lycopene), which lead to immunosuppression at cellular and humoral level during onset of lactation, consequently increasing susceptibility to infections [46,56]. Therefore, proper management of diet during transition period such as supplementation of vitamin E and zinc, is critical to prevent mastitis infection and to increase lactation [57,58].

### Environment factor

Environmental conditions and management practices of the herds have decisive effects on animal health and welfare. Keeping the herd clean and comfortable can reduce the incidence and severity of mastitis [59]. High stocking density, contaminated floor, wet bedding, poor ventilation, and hot and humid climate can promote growth of mastitis pathogens and increased exposure of cows, resulting in higher occurrence of mastitis [7,46,60].

## BOVINE MASTITIS CONTROL AND TREATMENT

### Five-point plan

Five-point plan introduced by National Institute for Research in Dairying (NIRD) since 1960s is effective in controlling contagious mastitis pathogens [61]. The five points are: i) identify and treat clinical cases; ii) post milking teat disinfection; iii) DCT; iv) cull chronic cases; v) routine maintenance of milking machine [14,62]. Unfortunately, the five-point plan is not very effective against the environmental pathogens and hence, is coupled with other appropriate strategies to control mastitis infections [63].

### Antibiotic therapy

The main strategy to treat mastitis is by the use of antibiotics, such as penicillin, ampicillin, tetracyclin, gentamycin, etc., which can be given by intra-mammary infusion, intramuscular or intravenous injections [64]. The DCT is one of the best choices to control and inhibit progression of mastitis. Dry period is an important stage in lactation cycle; any infection during dry period will affect the next lactation, and therefore, it is very important to take care of the cow’s health before the next milking cycle. Before drying off the cows, they were checked for any sign of mastitis; chronic mastitis cases, which are hard to detect by naked eyes, were checked via the California mastitis test (CMT) [65]. Then, right after the last milking, intra-mammary injection of antibiotic was applied to cow udder through canal teat, followed by application of teat sealant, which simulates the keratin plug, providing a physical barrier to bacterial invasion and preventing milk leakage. DCT can eliminate existing IMI and prevent new infection during dry period; thus, a dry cow tube consists of long persisting antibiotics, as they can deliver better cure rates [66,67]. An ideal treatment should be long enough to cure subclinical mastitis and short enough not to cause antibiotic resistance once the cow has calved. Dry cow period is the best time to cure mastitis; as there is no milk production during this period, the risk of incorporating antibiotic into the food chain is minimized, but caution should be taken even after calving [66].

Apart from this, any mastitis case detected during lactation is accompanied by a great concern of antibiotic residues in milk. When cow is detected with an active mastitis infection, the first thing to do is to cull the sick cow and milk out the cow completely to remove bacteria, milk clots, debris, and also toxins that might be released by the bacteria. Intra-mammary infusion of antibiotic is then applied for it to reach the udder as well as systematically into the blood circulation [64]. In some severe cases, where the inflammation is serious and the milk cannot be milked out completely, milk ducts will be blocked by milk debris, which will block the spreading of antibiotic throughout the udder. In that case, parenteral administration is advisable together with intra-mammary infusion on the advice of a veterinarian [64,68]. Long acting antibiotic is not suitable for mastitis detected during lactation, as getting the cow back to milking is the primary concern; therefore, it is important to gain knowledge about pathogen present in order to select appropriate cure for infected cows.

Despite the cost, the overuse and misuse of antibiotics in treating bovine mastitis has brought some problems to the dairy industry. In addition, the presence of antibiotic residues in milk is also of concern. In general, the milk obtained during the antibiotic treatment followed by a waiting period has to be discarded since it cannot be consumed by the consumer due to the risk of allergies and drug resistance caused by antibiotic residues [1]. Thus, heavy penalties are charged for antibiotic residues in milk. However, many drugs are still retained in the animal body for longer than the suggested discard times. So, the cost of treatment is determined by the loss incurred due to milk discarded rather than the cost of the drugs. Furthermore, even though antibiotics can eliminate infection, but they do not directly protect mammary gland from irreversible damage; farms are continuously experiencing loss due to decreased lifetime milk productivity [3].

### Vaccination

Vaccinating cows can be deemed as a preventive mastitis treatment in herds. Most vaccines are designed to target *Staph. aureus*, *Strep. agalactiae*, and *E. coli*. Vaccines targeting *Staph. aureus* and *Strep. agalactiae* are made up of either the whole organism (inactivated, high encapsulated or unencapsulated cells, and attenuated vaccines) or subunits (toxins, bacterial surface extract, and crude extract of polysaccharides); while for *E. coli*, the mutant core antigen J5 was used widely [69, 70]. However, vaccines are yet to provide reliable protection. For example, a widely available commercial vaccine named Startvac (Hipra SA., Girona, Spain) targeting *Staph. aureus* was studied in few reports. Schukken et al [71] demonstrated that Starvac can only moderately reduce new infection and duration of mastitis; whereas Bradley et al [72] reported that there was a significant reduction in severity of disease but increased milk production in Starvac-vaccinated cow when compared with non-vaccinated cow. Soon after that, Starvac was reported to be ineffective in improving udder health, milk production, or survival [73,74]. These varying degrees of vaccine efficacy might be associated with varying management practices of different herds [21].

As mentioned before, mastitis is caused by a number of different bacterial pathogens; therefore, the lack of efficacy of vaccines might also be due to the multi-etiological nature of bovine mastitis. Not only the site of infection in the mammary gland varies among different bacterial strains, but their virulence characteristics and immunogenic capabilities can also be different [75]. Hence, regardless of the type of vaccine, vaccination alone is not effective in preventing mastitis, especially in dairy herds that have high mastitis rates. Vaccination has to be coupled with other control procedures, such as hygienic milking, antibiotic treatment, infected cow culling, and so on, to reduce the incidence and duration of mastitis cases [69,71]. Indeed, it is necessary to find a vaccine that is able to protect against a wide range of strains since multiple strains can be present within a herd and within an individual cow [1]. It should also be easily implementable in the daily routine and be economically affordable [76].

## POTENTIAL ALTERNATIVES TREATMENT

As stated above, even though the use of antibiotics remains as the main treatment strategy, but its effectiveness is limited, not to mention the development of antibiotic-resistant strains of pathogen has become a critical challenge in antibiotic treatment [77,78]. Furthermore, the increasing concern of antibiotic resistance in public health issues is pushing the milk industries to reduce the usage of antimicrobial drugs. Therefore, seeking for alternative to antibiotic therapy, especially those derived from natural products such as plant and animal, is required [79–81].

### Plant-derived compounds

Plants have served as a valuable source for ingredients in traditional medicine therefore they are gaining interests of researchers in treating bovine mastitis. As compared to antibiotics, plant-derived compounds have an advantage of not inducing resistance even after prolonged exposure. Another advantage of plant-derived compounds is their low toxicity [82]. Various plants have been proved to exhibit antimicrobial properties and are also capable of inhibiting the inflammation induced by pathogens or endotoxin by inactivating NF-κB pathways. Antimicrobial activities against various pathogens were evaluated with paper disc assay, MIC assay, etc. *In vivo* mastitis studies were usually performed with BALB/c female mice, while *in vitro* anti-inflammation assays were usually carried out in primary bovine mammary epithelial cells (bMEC) or immortalized bMEC lines such as MAC-T and BME-UV1. The plant extracts along with their mechanisms of action are summarized in Table 1.

Baicalein is a flavone extracted from *Scutellaria baicalensis* and *Scutellaria lateriflora*, was claimed to attenuate inflammatory response by suppressing TLR4 mediated NF-κB and MAPK signaling pathways in LPS-induced mastitis in mice [83]. The binding of baicalein with glucuronic acid form a flavonoid glycoside named baicalin. Few studies had been carried out to study the effect of baicalin to combat bovine mastitis. Study performed by Zhao et al [84] showed that baicalin was able to inhibit *E. coli* strains isolated from mastitis milk samples with MIC of 4 mg/mL. Although the antibacterial activity of baicalin is low, it affects the drug resistance genes of *E. coli*, indirectly enhancing the sensitivity of *E. coli* to antimicrobial agents such as ampicillin, penicillin, streptomycin, ciprofloxacin. Before this, baicalin was shown to attenuate inflammation and apoptosis induced by *Staph. aureus* in mammary gland tissues of mouse model by regulating expression of pro-inflammatory cytokines and TLR2-related apoptotic factors [85,86]. Apart from that, Yang et al [87] also demonstrated that baicalin can attenuate LPS-induced inflammation and apoptosis of cow MEC, by inhibiting activation of NF-κB and up-regulation of heat shock protein 72.

Thymol, a monoterpene phenol isolated from thyme, oregano, and tangerine peel, has shown to have an anti-inflammatory effect on mouse mammary gland. It also reduces the expression of pro-inflammatory cytokines (TNF-α and IL-6) and inflammation mediated proteins iNOS and COX-2 in a dose-dependent manner, by down-regulating the activation of MAPK and NF-κB signaling pathway [88]. Besides, Wei et al [89] demonstrated that thymol can inhibit *Staph. aureus* internalization in bMEC in a dose-dependent manner. It was suggested that the reduction of *Staph. aureus* internalization was related to the inhibition of NF-κB nuclear translocation; however, this was not correlated with the ability of thymol to decrease the antimicrobial peptide gene expression such as tracheal antimicrobial peptide and β-defensin.

Resveratrol is a natural polyphenol that is present in various plant species, such as grapevines, berries, and peanuts. It has beneficial effects in protecting MAC-T from oxidative cell damage caused due to hydrogen peroxide [90]. Resveratrol was found to significantly inhibit mitochondrial-related cell death by down-regulating BCL-2-like protein 4 expression and up-regulating B-cell lymphoma 2 (BCL-2) expression. The detoxification of reactive oxygen species by resveratrol was proven through stimulation of several antioxidant defense genes such as heme oxygenase 1 (*HO-1*), cysteine/glutamate transporter, thioredoxin reductase 1 (*TXNRD1*), and NAD(P)H quinone dehydrogenase 1, which were up-regulated by activation of nuclear factor erythroid 2. In an *in vivo* study conducted by Zhang et al [91], resveratrol was able to inhibit LPS-induced mouse mastitis through the MAPK and NF-κB signaling pathways, suggesting that resveratrol can act as a potential therapeutic agent for mastitis.

Curcumin, the major compound of turmeric, was claimed to be one of the best potential therapeutic agent against bovine mastitis treatment [83]. Fu et al [92] injected curcumin 1 h before and 12 h after LPS treatment to mammary gland duct of mouse. They found out that it could attenuate the activity of myeloperoxidase, which was reflected by neutrophil accumulation in the mammary gland. The LPS-induced TNF-α, IL-6, and IL-1β were inhibited by curcumin through decreased expression of TLR4, and phosphorylation of IκBα and NF-κB p65. In addition, nanoformulation of curcumin showed even better effect in attenuating inflammatory responses induced by *Staph. aureus* in a mouse model when compared with normal curcumin [93]. In another study, the effect of turmeric on udder health of dairy cows was evaluated with a phytobiotics-rich herbal mixture (PRHM), which was made up of 18% turmeric roots, 18% cinnamon barks, 60% rosemary leaves, and 4% clove buds. Results showed that supplementation of PRHM were able to lower the SCC, especially in high SCC cows, demonstrating that PRHM could improve cow’s udder health. In addition, cows supplemented with PRHM also consumed more feed dry matter, which can improve feed utilization efficiency and produce a greater amount of milk, proving to be an effective strategy to enhance performance in cows afflicted with mastitis [94].

Despite having an effect on cell’s physiology, plant-derived compounds, especially essential oils, are reported to directly inhibit or kill mastitis pathogens (Table 2). Fratini et al [95] tested 10 commercial essentials oils on livestock mastitis-causing pathogens (*Staph. aureus*, *Staph. chromogenes*, *Staph. siuri*, *Staph. warneri*, *Staph. xylosus*, and *E. coli*) and found out that 3 of the essential oil *Satureja montana* L., *Thymus vulgaris* L. ct. thymol, and *Origanum majorana* L. were able to inhibit the tested pathogens. The study also found out that thymol, carvacrol and *p*-cymene are the most abundant components of these essential oils. Antimicrobial tests were carried out using both, pure component mixtures, as well as combined mixture of essential oils. In fact, the mixture of essential oils and mixture of pure components exhibited stronger inhibitory activity better than the single essential oil, suggesting there is synergistic effect between the mixtures. Fratini et al [96] further reported that the essential oil mixture of *Origarum vulgare* and *Leptospermum scoparium* may be an effective alternative to staphylococcal infections owing to their synergistic effect. In addition, Cho and his co-workers also found out that treatment using oregano essential oil (OEO) can improve physical condition of the udder in tested cows comparable to that by gentamycin. Not only SCC and the number of WBC were significantly decreased, but *Staph. aureus* and *E. coli* were not detected as well. This finding suggested that OEO might be an alternative to antibiotics in controlling subclinical bovine mastitis [97]. However, since OEO gave a distinct flavor and aroma to the milk samples of treated animals, long-term and higher dose exposure should be further investigated [98].

Terpeneless, cold-pressed Valencia orange oil was previ ously reported to have an antimicrobial effect on MRSA [99]. Federman et al [100] investigated the effects of citrus-derived oil (CDO) on the interaction between *Staph. aureus* and MAC-T cells. Growth of *Staph. aureus* was inhibited in a dose- and time-dependent manner. However only 0.05% CDO was found have a modest effect on the biofilm formation of *Staph. aureus*. When using MAC-T cells as an *in vitro* model of bovine mammary gland, 0.1% and 0.05% CDO managed to totally inhibit the adhesion and invasion of *Staph. aureus* into MAC-T cells. It was found out that major components of CDO, citral, and linalool are responsible for inhibition, owing to their abilities to alter the expression of *Staph. aureus* virulence genes [101]. On the contrary, CDO has also been reported to not impair the function of polymorphonuclear leukocytes, which play an important role in immune response against mastitis, at the same time inhibiting bacterial growth [102].

### Animal-derived compounds

Use of animal-derived compounds in treating bovine mastitis has been concentrated on bee products recently. Bee venom, containing the active component melittin, was administered to LPS-induced MAC-T cells to study its anti-inflammatory effect [103]. Authors found out that bee venom was able to attenuate the LPS-induced COX-2 protein expression, and also mRNA expression of pro-inflammatory cytokines TNF-α and IL-6, by down-regulating phosphorylation of ERK1/2 and nuclear translocation of NF-κB.

Propolis, a resinous substance produced by honey bee, has also been studied for its anti-inflammatory effect on MAC-T cells [104]. Pre-treatment of MAC-T cells with Chinese propolis (15 μg/mL) was able to prevent decrease in cell viability, as well as decrease in pro-inflammatory cytokines mRNA level such as TNF-α and IL-6, when stimulated with various pathogenic factors including LPS, lipoteichoic acid, TNF-α, heat-inactivated *E. coli*, and *Staph. aureus*. Besides, Chinese propolis also enhanced the mRNA expression of antioxidant gene *HO-1*, *TXNRD1*, and glutamate-cysteine ligase modifier subunit in mastitis infected cells, indicating the anti-oxidative effects of Chinese propolis.

On the contrary, immunomodulators naturally produced by mammals, such as lactoferrin, were preferred as potential non-antibiotic antimicrobial agents for treatment and prevention of bovine mastitis [1]. Lactoferrin is a multi-functional, iron-chelating glycoprotein found in milk, colostrum, and other exocrine secretions such as saliva and tears [105]. As an immunomodulator, it plays an important role in the innate immune system involving opsonization of microorganism for phagocytosis [46]. It was reported to exhibit antimicrobial effect against *E. coli*, *Pseudomonas aeruginosa*, *Strep. agalactiae*, and *Staph. aureus*, attributable especially to its iron-chelating ability, which can inhibit biofilm production through iron sequestration [105].

### Others

Other than bee products that are directly obtained from bee itself, lactic acid bacteria (LAB) found in the honey have been a new source of antibacterial agent [106]. LAB play an important role in honey production and protect the honey bees from different pathogens in hives and during nectar foraging [107]. Mixture of 13 species of LAB previously isolated from honey, from genera of *Lactobacillus* and *Bifidobacterium*, have shown to have an antibacterial activity on tested bovine mastitis isolates [106]. In fact, intra-mammary infusion of probiotics has emerged as a potential alternative in preventing and treating bovine mastitis, especially during dry-off period. *Lactococcus lactis* subp. *lactis* CRL 1655 and *L. perolens* CRL 1724 isolated from bovine milk can inhibit bovine mastitis pathogens. These species were able to adhere to teat canal, therefore hypothesized to have a role in prevention of bovine mastitis during dry period [108]. Apart from the milk, LAB isolated from bovine mammary microbiota also exhibit beneficial properties to udder. Nine of the LAB species isolated exhibited anti-inflammatory response in bMEC stimulated by *E. coli*. In addition, both *L. brevis* 1595 and 1597 and *L. plantarum* 1610 showed high colonization capacities towards bMEC, suggesting they can be good candidates to compete with pathogens in mammary gland colonization [109].

Bacteriocins, antimicrobial peptides produced by bacteria, have emerged as potential alternative for bovine mastitis [110, 111]. One of the most studied bacteriocin in bovine mastitis is nisin, which is a lantibiotic, containing 34-amino acid residues, produced by *L. lactis*. Nisin form a complex with the cell wall, thereby inhibiting cell wall biosynthesis. The complex then aggregates and further incorporates into the cell wall, finally forming a pore in the bacterial membrane [112]. Nisin is used as an active agent in teat wipe named Wipe Out [113], however, *Staphylococci* were reported to have nisin resistance, therefore, discovery of new bacteriocins, alone or in combination with nisin, are highly desirable [114]. Field et al [115] reported that nisin derivatives in combination with antibiotics, namely, nisin V and I4V, significantly increased biofilm inhibition activity against *Staph. aureus* and *Strep. pseudintermedius* than wild-type and antibiotics combination. Besides, combination of nisin and dioctadecyldimethylammonium bromide nanoparticles increased the susceptibility of *Staphylococci* to nisin [116]. Another bateriaocin, lysostaphin, isolated from *Staph. simulans*, either treated alone or in combination with nisin, can inhibit biofilm-forming *Staph. aureus* [117]. Taken together, combination of nisin with other antimicrobial agent can overcome the issue of nisin resistance.

Bacteriophage are viruses that specifically infect bacteria and are harmless to humans, animals, and plants; thus, bacteriophage and their derivatives (i.e., endolysin, exolysin, and depolymerase) are being deemed as valuable antimicrobial alternatives with a potential to reduce the current use of antibiotics in agri-food production, increasing animal productivity and providing environmental protection [118]. Varela-Ortiz et al [119] isolated 4 phage lysates from an apathogenic *Staph. aureus* strain and tested on 36 *Staph. aureus* subclinical mastitis strains. They found out that all the tested strains were susceptible to all phage lysates. Other than *Staph. aureus*, bacteriophage was reported targeting *E. coli* too. Porter et al [120] separated bacteriophages from 36 clinical coliform mastitis isolates and selected 4 phages in combination with distinct broad host range as candidates to evaluate its antibacterial activity against mastitis-causing *E. coli*. Bacteriophage cocktail had same effect as ceftiofur (10 μg/mL) in inhibiting *E. coli* growth. It also significantly reduced adhesion and invasion of *E. coli*. In addition, combination of bacteriophage with a non-antibiotic bismuth-based intra-mammary teat sealant, *E. coli* growth was inhibited, therefore, phage cocktail was suggested to have a potential to control *E. coli* infections in farm [120]. Moreover, bacteriophages also showed the potential to be suitable for vaccination when engineered with genes of interest, thereby, can be useful against bacterial and viral infections [118]. For instance, treatment using recombinant endolysin Trx-SA1 to mild clinical *Staph. aureus* mastitis quarters showed significant reductions in pathogen levels and SCC [121].

Chitosan is a natural polysaccharide derived from chitin, proven to have broad spectrum of antimicrobial activity against fungi and bacteria. It was majorly reported to inhibit growth and biofilm formation of *Staphylococcus* spp. causing bovine mastitis [27,122]. Chitosan in nanoparticle form exhibit higher antimicrobial and anti-biofilm capacity than the native chitosan [123]. Intra-mammary infusion of chitosan can boost up the mammary gland involution and activate host innate immunity, associated with an increase in SCC, bovine serum albumin and lactoferrin concentrations. It also increases lactate dehydrogenase activity in mammary secretions, which consequently reduce the possibility of getting new IMIs during the dry period [124].

## CONCLUSION

In conclusion, effective mastitis control programs rely more on prevention rather than treatment. Currently, antibiotic treatment is still an established component in mastitis control programs. Antibiotics are often coupled together with others therapies; yet the effectiveness is still not satisfying. Therefore, searching for new therapeutic alternatives is necessary. A wide variety of natural products derived from plants, animals, and bacteria were investigated and reported to have potential in controlling bovine mastitis. Field studies should be considered to reassure the outcome of the alternative therapies before commercial applications.

## Figures and Tables

**Table 1:** Recent studies on plant-derived compounds against bovine mastitis

Plant/Extract	*In vitro/in vivo* model	Stimulant	Mechanisms of action
Baicalein [83]	BALB/c mouse	LPS	Attenuate inflammation by suppressing activation of TLR2, TLR4, NF-κB, and MAPK signaling pathway
Thymol [88]	BALB/c mouse	LPS	
Resveratrol [125]	BALB/c mouse	LPS	
Bergenin [126]	BALB/c mouse	LPS	
Leonurine [127]	BALB/c mouse	LPS	
Curcumin [92]	BALB/c mouse	LPS	
Luteolin [128]	BALB/c mouse	*Staph. aureus*	
Puerarin [129]	BALB/c mouse	*Staph. aureus*	
Baicalin [85]	BALB/c mouse	*Staph. aureus*	
Baicalin [87]	Primary bMEC	LPS	
Morin [130]	Primary bMEC	LPS	
Forsythoside A [131]	Primary bMEC	*Staph. aureus*	
Phytoncide [132]	MAC-T	LPS	
*Moringa oleifera* leaf extract [133]	MAC-T	LPS	
Resveratrol [90]	MAC-T	H_2_O_2_	
Baicalin [86]	BALB/c mouse	*Staph. aureus*	Inhibits apoptosis by regulating TLR2, BCL-2, BAX, and caspase-3
Thymol [89]	bMEC	*Staph. aureus*	Inhibits *Staph. aureus* internalization by inhibiting NF-κB activation
Citral and linalool [101]	MAC-T	*Staph. aureus*	Inhibits *Staph. aureus* growth and biofilm formation, reduced adhesion and invasion in MAC-T by altering expression of the virulence genes
Baicalin [84]	N/A	N/A	Antimicrobial action against *E. coli*, damage bacteria cell wall and affecting the drug resistance genes of *E. coli*, reduce antimicrobial agents’ resistance
Limonene [134]	N/A	N/A	Antimicrobial activity against *Strep. uberis*
*Liquidambar orientalis* leaf extracts [135]	N/A	N/A	Antimicrobial activity against *Staph. aureus* and *Coagulase Negative Stapylococci*
*Thalictrum minus* root extract [136]	N/A	N/A	Antimicrobial activity against *Staph. xylosus, Staph. lentus, Staph. equorum, Enterococcus faecalis*, and *Pantoea agglomerans*
*Eucalyptus globulus* leaf extract and *Juglans regia* plant extract [137]	N/A	N/A	Inhibits *Staph. aureus* growth and biofilm formation
*Terminalia chebula* fruit extracts [138]	N/A	N/A	Antimicrobial activity against *Staph. aureus, E. coli, Pseudomonas aeruginosa*, and *Bacillus megaterium*
*Poncirus trifoliate* fruit extract [139]	N/A	N/A	Antimicrobial activity against *E. coli, Haemopillus somnus, Burkholderia* spp., *Haemopillus parsuis, Clostridium perfringens*, and *Pantoea agglomerans*

LPS, lipopolysaccharide; TLR, toll-like receptor; NF-κB, nuclear factor kappa B; MAPK, mitogen-activated protein kinase; MAC-T, bovine mammary alveolar cells; BCL-2, B-cell lymphoma 2; BAX, BCL-2-like protein 4; bMEC, bovine mammary epithelial cells.

**Table 2:** Recent studies on plant essential oils against bovine mastitis

Essential oil origin	Mechanisms of action
Mixture of *Satureja montana* L., *Thymus vulgaris* L. ct. thymol, and *Origanum majorana* L. [95]	Antimicrobial activity against *Staph. aureus*
Mixture of *Origanum vulgare* and *Leptospermum scoparium* [96]	Antimicrobial activity against *Staph. aureus, Staph. chromogenes, Staph. siuri, Staph. warneri, Staph. xylosus* and *E. coli*
*Origanum vulgare* [97]	Decreased SCC and WBC in cows afflicted with subclinical mastitis, inhibits *Staph. aureus* and *E. coli*
Valencia orange [100]	Inhibits *Staph. aureus* growth and biofilm formation, reduced adhesion and invasion in MAC-T
*Minthostachys verticillata* and Citrus [134]	Antimicrobial activity against *Strep. uberis*
*Minthostachys verticillata* [140]	Attenuate *Entero*. faecium-induced inflammation in mammary gland tissue of mouse model by activating macrophage phagocytosis and modulating innate immune response
*Cinnamon cassia* [141]	Antimicrobial activity against *Staph. aureus, Staph. epidermidis, Staph. hyicus, Staph. xylosus*, and *E. coli* 29
Patchouli, Cedar, Thyme, and Manuka [142]	Antimicrobial activity against *Staph. aureus, Staph. epidermidis*, and *Staph. xylosus*

SCC, somatic cell count; WBC, white blood cell; MAC-T, bovine mammary alveolar cells.
